# The multi-level outcome study of psychoanalysis for chronically depressed patients with early trauma (MODE): rationale and design of an international multicenter randomized controlled trial

**DOI:** 10.1186/s12888-023-05287-6

**Published:** 2023-11-16

**Authors:** Gilles Ambresin, Marianne Leuzinger-Bohleber, Tamara Fischmann, Nikolai Axmacher, Elke Hattingen, Ravi Bansal, Bradley S. Peterson

**Affiliations:** 1 Department of Psychiatry-CHUV, University Institute of Psychotherapy, The University of Lausanne, Lausanne, Switzerland; 2 University Medical Center of the Johannes Gutenberg University Mainz, Mainz, Germany; 3 International Psychoanalytic University, Berlin, Germany; 4 Research Department of Neurosciences, Ruhr University, Bochum, Germany; 5 Department for Neuroradiology, University Hospital, Frankfurt, Germany; 6 Department of Pediatrics, Keck School of Medicine at the University of Southern California, Los Angeles, CA USA; 7 Institute for the Developing Mind, Children’s Hospital Los Angeles, Los Angeles, CA USA; 8 Department of Psychiatry at the Keck School of Medicine, University of Southern California, Los Angeles, CA USA

**Keywords:** Chronic depression, Early trauma, Psychoanalytic psychotherapy, Psychoanalysis

## Abstract

**Background:**

Whether and how psychotherapies change brain structure and function is unknown. Its study is of great importance for contemporary psychotherapy, as it may lead to discovery of neurobiological mechanisms that predict and mediate lasting changes in psychotherapy, particularly in severely mentally ill patients, such as those with chronic depression. Previous studies have shown that psychoanalytic psychotherapies produce robust and enduring improvements in not only symptom severity but also personality organization in patients who have chronic depression and early life trauma, especially if therapy is delivered at a high weekly frequency.

**Methods/design:**

Patients with chronic major depression and a history of early life trauma will be recruited, assessed, and treated across 3 international sites: Germany, Switzerland, and the United States. They will be randomized to one of two treatment arms: either (1) once weekly psychoanalytic psychotherapies, or (2) 3–4 times weekly psychoanalytic psychotherapies. They will have full clinical characterization as well as undergo MRI scanning at study baseline prior to randomization and again one year later. A group of matched healthy controls will undergo similar assessments and MRI scanning at the same time points to help discern whether study treatments induce brain changes toward or away from normal values. Primary study outcomes will include anatomical MRI, functional MRI, and Diffusion Tensor Imaging measures. Study hypotheses will be tested using the treatment-by-time interaction assessed in multiple general linear models with repeated measures analyses in an intent-to-treat analysis.

**Discussion:**

MODE may allow the identification of brain-based biomarkers that may be more sensitive than traditional behavioral and clinical measures in discriminating, predicting, and mediating treatment response. These findings could help to personalize care for patients who have chronic depression patients and early life trauma*,* and they will provide new therapeutic targets for both psychological and biological treatments for major depressive illness.

**Supplementary Information:**

The online version contains supplementary material available at 10.1186/s12888-023-05287-6.

## Introduction

### Chronic depression and psychoanalytic psychotherapy research

Chronic depression is a common disorder. Population lifetime prevalence estimates are 3–15% for major depression [1–6] and 3–4% for dysthymia [7–10], with 20–33% of all depressive illness becoming chronic [3, 11]. Persons whose depression is chronic tend to have more severe symptoms, more frequent recurrences, lower remission rates, longer courses of medication treatment [12, 13], and higher rates of psychiatric and physical co-morbidities [2, 3, 12, 14] compared with depressive episodes of shorter duration. Recent epidemiological findings suggest that chronic depression is associated with a series of potentially modifiable risk factors, such as increase in suicidal ideation/attempts during the most severe episode, higher levels of neuroticism, lower levels of extraversion and lower levels of informal help-seeking behavior compared to nonchronic MDD, that are accessible via psychotherapies that can improve the course of chronic depression [15]. Chronic depression entails enormous direct and indirect costs for health care systems (see e.g. [16]).

Patients with chronic depression require tailored psychotherapeutic treatments that address their specific clinical needs [17]. Meta-analyses show that various forms of short-term therapy are similarly successful in reducing symptom severity but do not significantly reduce relapse rates [11, 18–27]. Increasing evidence suggests that these patients require more intensive and longer treatments to achieve sustained symptomatic and functional improvement [17, 28–31].

Only a small number of studies, however, have assessed the effectiveness of long-term therapies for chronic depression [24, 25, 32–40]. We refer to two more recent studies that assessed the effectiveness of long-term psychoanalytic psychotherapy for chronic depression in particular. One was The Tavistock Adult Depression Study (TADS), which was a randomized controlled trial of 129 patients with chronic depression of at least 2 years duration. It assessed the effectiveness of 18 months of long-term psychoanalytic psychotherapy (LTPP) as an adjunct to treatment-as-usual (TAU), compared to TAU alone. The TAU group was treated with interventions as directed by the referring practitioner, which could include referral to other specialists in accordance with UK guidelines of the National Institute for Clinical Excellence (National Institute for Health and Clinical Excellence [41]. Both clinician- and self-reported depression severity, as well as measures of social adjustment, showed significantly greater improvement in the LTPP group [39].

The second was the LAC Study (Langzeitbehandlungen **c**hronisch depressiver Patienten), which compared the effectiveness of long-term cognitive-behavioral therapy (CBT) vs long-term psychoanalytic therapy (PAT) in 252 chronically depressed patients. The study also considered the effects of preferential or randomized allocation [42]. As is the practice of the two forms of psychotherapeutic interventions in Germany, the number of sessions offered to the patient varied. During the first year of treatment, CBT patients received a mean (SD) of 32.5 (9.0) therapy sessions while PAT participants received a mean (SD) of 80.4 (27.8) sessions. Both psychotherapies and both allocation conditions showed a clinically relevant and highly significant reduction of depressive symptoms over 1, 2, and 3 years. After 3 years, self-rated full remission rates were 45% (BDI ≤ 12), and clinician-rated full remission rates were 61% (QIDS-C ≤ 5). The effect sizes were very large (d = 1.78 for the BDI); d = 2.12 for the QIDS-C). These effect sizes and full remission rates were better compared to other studies [11, 29, 34]. As in other studies, researchers found no significant difference between PAT and CBT for symptom reduction 3 years after beginning treatment.

In contrast to the original hypotheses PAT produced a statistically significant reduction in depressive symptoms already after one year of treatment. In addition, three years after the start of treatment, significantly more patients in psychoanalytic treatment showed increased awareness of unconscious fantasies and conflicts, so-called “structural changes, in personality organization compared to patients in cognitive-behavioral treatments [30]. Clinical observations confirmed that these changes were more pronounced in high-frequency treatments than in low-frequency treatments.

### Early trauma, frequency of sessions, and treatment effects in chronic depression

An unexpected, post hoc finding of the LAC study was that more than 80% of chronically depressed patients participating in the study suffered from early trauma, and that these patients responded especially well to high frequency psychoanalytic psychotherapy, often achieving sustained, structural changes in personality organization. A review of clinical observations revealed that patients in high-frequency psychoanalytic treatments were more likely to be successful in therapeutically regaining the primal trust in a helping Other and their own self agency that had collapsed as a result of traumatization than in low frequent treatments (see e.g. [43–46]; or “epistemic trust” – see [47]. These clinical effects were connected to some extent to "structural changes" in personality organization as assessed using the Operationalized Psychodynamic Diagnosis (OPD) [29, 30, 47–49]. This clinical finding was consistent with a previous study showing that chronically depressed patients who had a history of early childhood trauma achieved significantly higher remission rates with psychotherapy monotherapy than with nefazodone monotherapy [50], leading to the conclusion of the study investigators that chronically depressed patients who have a history of early trauma may differ in some fundamental yet undefined way from those without a history of early trauma [50]. A meta-analysis [51] also showed that neglect and emotional abuse are correlated with adult depression. In the LAC Study emotional neglect, as measured by the CTQ, was the most frequent form of childhood trauma of the chronic depressed patients and influenced the severity of the depression in this group of patients (see e.g. [52]).

A meta-analysis of including a variety of psychotherapies for chronic depression reported a strong association for symptomatic improvement with the number of treatment sessions [53]. Moreover, both a higher number of weekly psychoanalytic sessions, as well as a more pronounced application of psychoanalytic treatment techniques, increased the effectiveness of psychotherapy in patients with major depression [54]. Session frequency has also been identified as an important factor in the efficacy of psychotherapy in general. An overview focusing on the difference between psychoanalysis and psychoanalytic psychotherapy suggested that the success of therapy does not correlate most with the duration of the treatment, but with the frequency of sessions [31]. These findings suggest that a higher treatment frequency is more effective than lower treatment frequency in reducing symptoms. In sum, findings suggest that higher session frequency may offer an advantage that goes beyond the total number of sessions in the psychoanalytic psychotherapy of depression. Still unknown is whether a similar frequency effect is present in the psychoanalytic treatment of depression that is chronic.

### Psychotherapy research in depression using brain imaging

Several investigators have used brain imaging technologies to study the associations of psychotherapies with measures of brain structure and function [55], including in depressed patients [56–66]. Lueken & Hahn [67] describe two general approaches that have been employed thus far: 1) the longitudinal study of brain changes, comparing pre- to post-treatment brain measures, associating those changes with measures of treatment response to psychotherapy; and 2) associating pre-treatment brain measures with treatment outcomes, such as depressive symptom reduction, to identify brain predictors of treatment response. Most of these studies have been naturalistic (without employing randomization between treatment arms) and they often have been uncontrolled (without a comparison group). Three meta-analyses summarized studies on treatment effects on clinical and brain-based predictors [68–70]. Cristea et al. [71] combined a meta-analysis with a review of biological markers for psychotherapy in treating depression. They found limited evidence that the benefits of psychological treatments for depression translate to biological outcomes, in that only neuroimaging markers showed promise in predicting treatment response. Two neuroimaging studies are particularly important for MODE: The Hanse-Neuro-Psychoanalysis Study investigated unmedicated outpatients with recurrent depression (*n* = 16) and healthy controls (*n* = 17) before and after 15 months of 2 or more sessions of weekly psychoanalytic psychotherapy. In the fMRI study, neural activity associated with the processing of personalized attachment material changed in patients from baseline to endpoint, but not in healthy controls. The Frankfurt fMRI/EEG Depression Study (FRED) studied a subsample of participants in the LAC Depression study (see 2.1.2, *n* = 24 and 24 controls) using the Operationalized Psychodynamic Diagnosis (in analogy to the Hanse-Neuro-Psychoanalysis Study) and dream words taken from a significant dream elicited in a dream interview. Measurements were obtained at three different time points, each one a minimum of 8 months apart. Results of the dream experiment revealed that dream words, in contrast to neutral words, showed a differential activation of medial orbitofrontal areas, functionally related to the self and self-agency. Results of the OPD Experiment – using the OPD-sentences collected previously – yielded similar conclusions (see Fischmann et al. [72–75]).

## Objectives

Although considerable evidence supports the clinical effectiveness of psychoanalytic therapies, we do not yet understand the mechanisms through which they improve symptoms and reduce functional impairments – knowledge that can further enhance treatment effectiveness, especially in the highest risk and most complex clinical populations. This mechanistic clinical trial will identify the mediators of psychoanalytic therapy treatment response in depressed patients. These neurobiological as well as clinical mediators, once identified, will allow their targeting in the development of new therapies and in improving existing ones. Using these mediators as surrogate endpoints will facilitate smaller and more cost-effective future clinical trials of psychoanalysis than those that have only clinical measures as endpoints [76]. These aims are consistent with the NIMH experimental therapeutics initiative to identify mechanistic targets for clinical trials development [77–79].

In addition to identifying mediators of treatment response, this mechanistic clinical trial will identify baseline predictors of psychoanalytic treatment response in chronic, severely depressed patients. In future studies, we will be able to use the predictors we discover here to determine, for example, whether we can screen depressed patients for these predictive biomarkers and thereby significantly improve treatment response rates. Identifying these markers will provide a framework for developing personalized care in depressed patients.

The prototypical study design in modern medicine for inferring causation is the Randomized Controlled Trial (RCT), which assesses the effects of an experimentally manipulated intervention on the dependent (outcome) variable, usually illness severity. RCTs studying the efficacy and effectiveness of psychotherapy traditionally use clinical measures of symptom change as their primary outcome measure. Many cognitive, behavioral, and neural systems influence symptom severity, however, making these outcome measures very noisy, which in turn reduces the statistical power to detect real change that the therapies produce. Low statistical power in turn necessitates large numbers of participants to detect significant effects of the intervention, thereby increasing the size, duration, and cost of RCTs. Instead of employing change in symptom severity as the usual clinical outcome measure, MODE will use the change in brain imaging measures from the study baseline to study endpoint as the primary study outcome, providing strong causal inference that the treatment causes the changes in brain measures and depressive symptoms observed [80]. In our prior studies using these technologies, brain measures compared with traditional clinical measures provided much more sensitive and robust markers for treatment response, because brain measures are direct reports that the treatments have on the brain, which in turn underlie the change in symptom severity. MODE will be therefore identifying the biomarkers that predict and mediate change in symptoms and other clinical outcomes in high vs low frequency of psychoanalytic therapy in severely and chronically depressed patients.

### Specific aims and hypotheses

#### AIM 1

Identify how long-term psychoanalytic therapies change brain structure and function in chronically depressed patients with a history of early trauma and to assess how those changes differ in patients assigned randomly to either high or low frequency weekly sessions.

#### AIM 2

Assess whether these brain changes significantly mediate clinical improvement over 1 year of treatment.

#### Primary hypotheses

High compared with low frequency treatment will normalize brain structure and function.

##### Brain structure

Based on prior findings in chronic depression patients in an RCT of medication therapy vs placebo [81], changes in cortical thickness, representing therapy-induced, neuroplastic compensatory changes in brain structure, will normalize during 1 year of treatment – i.e., baseline abnormalities relative to healthy control values will change more during high frequency treatment compared with low frequency treatment, and 1 year after beginning treatment will be statistically indistinguishable from healthy control values. Change in cortical thickness will correlate significantly with the magnitude of clinical improvement after 1 year of treatment.

##### Brain activation

High compared with low frequency treatment will produce greater change in brain activity (a greater degree of normalization relative to healthy control values) in ventral medial brain regions during the functional MRI (fMRI) Cyberball paradigm for social exclusion, and the changes in brain activity will significantly mediate clinical improvement during the 1-year duration of treatment.

##### Functional connectivity

High compared with low frequency treatment will produce a greater change in measures of functional brain connectivity after 1 year of treatment during resting state fMRI, particularly in the default mode network (DMN), which likely subserves the capacity for free association. Based on prior findings in chronically depressed patients in an RCT of medication therapy vs placebo [82], we expect high compared with low frequency treatment to produce a greater degree of normalization in DMN activity relative to activity in healthy control participants.

##### Tissue microstructure

High compared with low frequency treatment will produce a more pronounced normalization of indices of tissue microstructure in white matter (increasing fractional anisotropy and decreasing mean diffusivity) measured with diffusion tensor imaging (DTI).

#### Secondary hypotheses

##### Psychopathology

Although high and low frequency treatments will both produce clinical improvement, improvement will be significantly greater for high frequency treatment in structural integration (measured with the Operationalized Psychodynamic Diagnosis Structure Questionnaire (OPD-SQ)), depressive symptoms (measured with the BDI-2), and in the manifest dreams (measured with DREams Assessment Scale for measuring Changes in manifest dream (DREAMS-C). Improvement in these clinical measures will associate significantly with the regional changes in MRI measures that change in response to high vs low frequency treatment.

##### Metabolic profile (only for a subgroup in Frankfurt)

Proton MR Spectroscopy (^1^H-MRS) measures brain metabolites N-acetyl-aspartate (NAA), choline (Cho) and creatine (Cr) as potential in vivo biomarker of the integrity of neurons (NAA), membranes (Cho) and glia (Cr) [83, 84]. High compared with low frequency treatment will produce a more pronounced changes of metabolite concentration in N-Acetylaspartate (NAA/Cr, respectively), indicating normalization of neuronal integrity measured with MR spectroscopy (^1^H MRS). Change in NAA (NAA/Cr) will correlate significantly with the magnitude of clinical improvement after 1 year of treatment.

## Methods/Design

### Study design

**Fig. 1:**
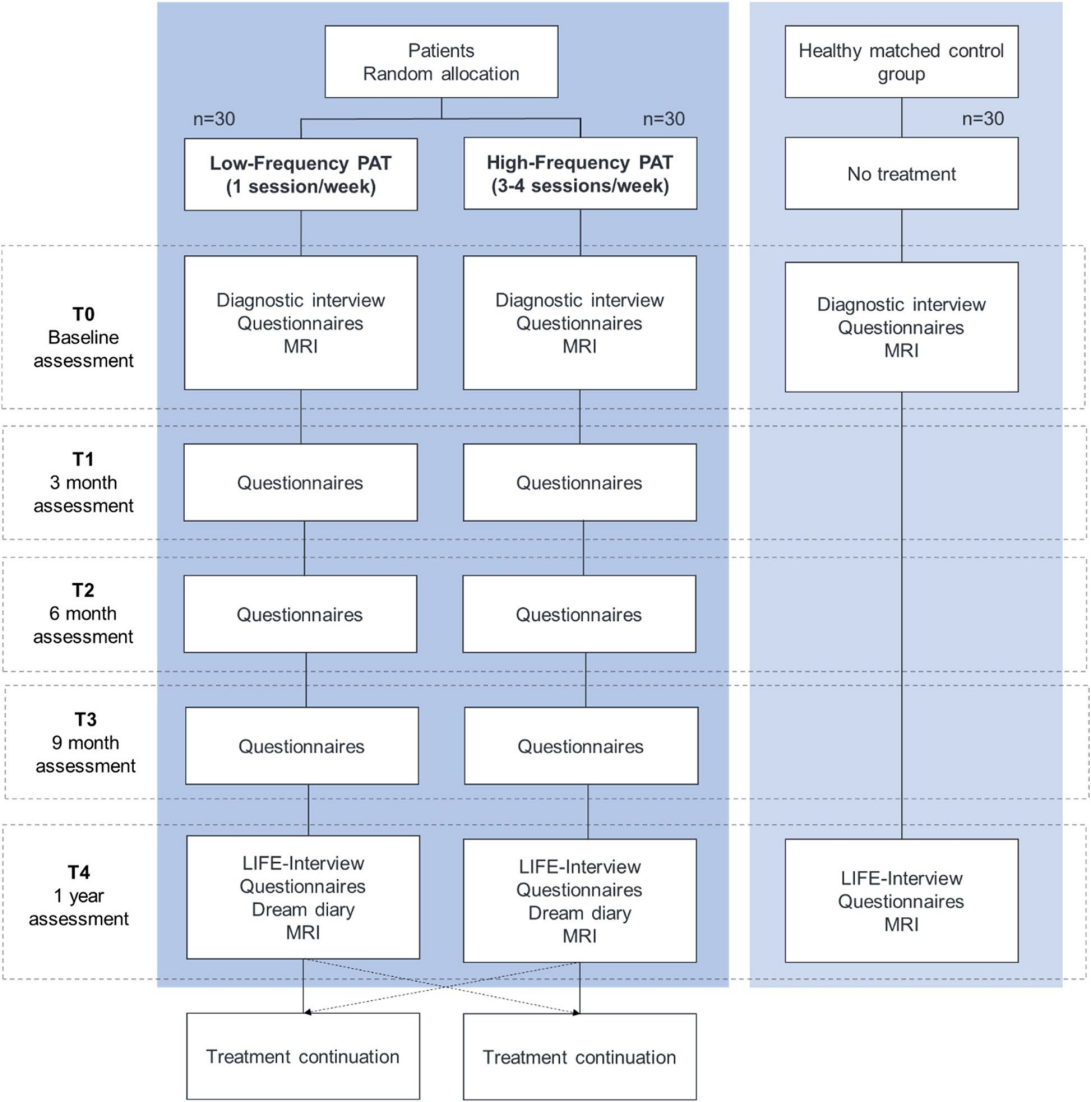
Flow chart of the study

MODE is a randomized, parallel, superiority and single blinded controlled trial comparing the effectiveness of two active interventions – high frequency (*n* = 30) vs low frequency (*n* = 30) psychoanalyses – in modifying brain structure and function over one year of treatment chronic depression patients who have a history of early life trauma (Fig. 1).

This study design will overcome many of the limitations of prior psychotherapy effectiveness studies. It will include an active control condition rather than a mere wait list, thereby controlling for the effects of interpersonal attention and interaction. Randomization and statistical evaluation will be performed by independent teams. Moreover, the active comparator condition, low frequency psychoanalysis, will help to control for the “allegiance” effects that confound most psychotherapy trials [85]. Finally, comparing high vs low frequency psychoanalyses will inherently provide an assessment of dose–response effects of psychoanalytic therapy on brain structure and function. Establishing dose–response associations will further improve causal inference in our study design.

In addition to randomization of depressed patients, we will include a group of healthy control participants (*n* = 30) who are free of psychiatric illness. They will not undergo treatment and therefore will not be randomized. They will undergo similar assessments and MRI scanning at one-year intervals. Their measures will serve as reference points to help discern whether study treatments induce brain changes toward or away from normal values.

### Inclusion and exclusion criteria

#### Patients

Patients between 21 and 60 years of age of all genders suffering from chronic depression will be included in the study. Patients must be continuously depressed for more than one year and currently meet a diagnosis of major depression or dysthymia. The SCID interview will confirm a yearlong history of depression. Only patients without antidepressant (AD) medication at study onset will be included. Only patients with early trauma on at least one of the subscales of the CTQ will be included (Table 1).

**Table 1:** Inclusion and exclusion criteria for patients

Inclusion Criteria	Exclusion Criteria
• Diagnosis of major depression and/or dysthymia (based on Structural Clinical Interview-SCID) for at least 12 months • BDI-2 > 17 • QIDS > 9 • CTQ: at least trauma at one of the subscales • Age: 21–60 years • Sufficient knowledge of local language • Informed consent to study protocol • Need to be free of anti-depressant, anti-anxiety, and antipsychotic medication for at least 3 weeks before baseline assessment and be deemed stable by their treating clinician at the study onset	• Current or past psychotic symptoms in the last 3 years, schizoaffective, schizophrenic, or bipolar affective disorder • Substance dependence current or during the last three years • Dementia • Borderline, schizotypal, or antisocial personality disorder • Acute suicidality • Reduced intellectual capacity • Serious physical illness that strongly affects the depression or is causal for the depression • Concurrent psychotherapy • Technical exclusion criteria for MRI (metallic tattoos, pacemakers, or other metal parts in the body etc., according to special guidelines of the local MRI team)

#### Healthy control participants

Inclusion criteria are: (1) no history of prior or current psychiatric diagnoses (Structured Clinical Interview for DSM-SCID); (2) no prior history of trauma on any of the CTQ subscales; (3) 21–60 years of age; and (4) sufficient knowledge of the local language to undergo assessments. Exclusion criteria are: (1) serious physical illness; and (2) any current psychotherapeutic or psychotropic treatment. Healthy controls will be group-matched on age and gender to participants in the treatment groups (Table 2).

**Table 2:** Inclusion and exclusion criteria for healthy participants

Inclusion Criteria	Exclusion Criteria
• No history of prior or current psychiatric diagnoses (based on Structural Clinical Interview-SCID) • CTQ: No prior history of trauma on any of the subscales • Age: 21–60 years • Sufficient knowledge of local language • Informed consent to study protocol	• Serious physical illness that strongly affects the depression or is causal for the depression • Concurrent psychotherapy • Technical exclusion criteria for MRI (metallic tattoos, pacemakers, or other metal parts in the body etc., according to special guidelines of the local MRI team)

#### All participants

Standard MRI exclusion criteria will apply (metallic tattoos, pacemakers, or other metallic implants).

### Intervention and comparator

Study intervention will be comprised of low frequency psychoanalytic psychotherapy (1 session a week) for early traumatized, chronically depressed patients and high frequency psychoanalytic psychotherapy (3–4 weekly sessions). The active comparator condition, low frequency psychoanalysis, delivered by study therapists who are experts in delivering both frequencies, and with the same overall manual based theoretical framework and technical interventions [86], will ensure that they will be equally aligned to both high and low frequency therapies. A group of healthy controls will be needed for brain imaging comparison purposes. A control group will allow to measure cortical thickness in patients with chronic depression in comparison to healthy control participants. Therapy sessions (45–50’ each) will be delivered in the private practice rooms or rooms of the outpatient clinics of the local psychotherapeutic institutions. Patients who will require a combination therapy of medication and psychotherapy during treatment will not be excluded but form a special subgroup that will be treated separately during data analyses. Patients who will require a change in psychotherapy setting during treatment according to patient and therapist decision will be analyzed as randomized. MODE study duration will be one year, but after 1 year of treatment initiation, treatment may continue according to patient and therapist decision. Treatments will be financed in the usual way in the participating countries (in Germany by the health insurance companies, in the US and Switzerland partly by the health insurance companies, partly by the patients themselves).

### Outcome measures

Study participants will be assessed as detailed below and in Table 3, for primary and secondary outcome measures. Patients will be assessed by independent and trained clinicians who are blind to treatment conditions.

**Table 3:** Study timetable of assessments

Assessments	Screening	Visit 1	Visit 2	Visit 3	Visit 4	Visit 5
*Description*	*Inclusion*	*Study start*	*FU 3 months*	*FU 6 months*	*FU 9 months*	*Study end*
*Days*	*Day -x*	*Day 0*	*Day 91*	*Day 182*	*Day 273*	*Day 365*
*Window (dates)*			± *7*	± *7*	± *7*	± *3*
*Weeks*		*0*	*13*	*26*	*39*	*52*
Informed consent signed	X					
Eligibility	X					
Demographics		X				
SCID (CI)	X	(X)^a^				X
Psychoanalytic interview	X	(X)^a^				
MRI scan	X	(X)^a^				X
Trust game	X	(X)^a^				X
GAF (Cl)		X				X
BDI-II (SR)	X	X	X	X	X	X
DEQ (SR)		X	X	X	X	X
QIDS (Cl, SR)		X	X (only SR)	X (only SR)	X (only SR)	X
SCL-90-R (SR)		X	X	X	X	X
CTQ (SR)	X					
IIP (SR)		X	X	X	X	X
Work Ability Index (SR)		X	X	X	X	X
OPD_SF (SR)		X	X	X	X	X
Dream Diary (SR)						X
Patient trauma narratives		X				X
WAI-SRp (SR)		Collected each month from Assessment 1 to 5				
Psychotherapy		Throughout study				
Adverse Events			X	X	X	X
SAE		Throughout study				
Medication		X	X	X	X	X

*FU* Follow-up, *Cl* Clinician rated, *SR* Self-reported, *SCID* Structured Clinical Interview for DSM, *GAF* Global Assessment of functioning, *BDI-II* Beck Depression Inventory-II, *DEQ* Depressive Experience Questionnaire, *QIDS* Quick Inventory of Depressive Symptoms (Cl, SR), *SCL-90-R* Symptom Checklist, *CTQ* Childhood Trauma Questionnaire, *IIP* Inventory Interpersonal Problems, *WAI* Work Ability Index, *OPD-SF* OPD structure questionnaire, *WAI-SRp* Working Alliance Inventory, *SAE* Severe Adverse Event

^a^Depending on the availability of the persons concerned, the first MRI examination, the administration of the Trustgame, the SCID interview, and the psychoanalytical interview can take place at the screening visit or visit 1

#### Primary outcomes: MRI brain measures

Assessment will comprise the following MRI brain measures. Anatomical MRI: measures of cortical thickness at all points across the cerebrum. Resting State Functional MRI (rsfMRI): measures of functional connectivity throughout the brain. Cyberball Task-Related Functional MRI (fMRI): measures of brain activation throughout the brain in response to trust, mistrust (social exclusion), and rebuilding of trust (social re-inclusion). Diffusion Tensor Imaging (DTI): measures of fractional anisotropy and average diffusion coefficient.

What these outcomes tell us about the brain MRI is an entirely safe way of imaging the brain at any age. It is routinely performed in unsedated people at multiple time points, including multiple time points within an RCT, and without the need for exposure to any radioactivity. MRI scanning can be performed in different modes, with each modality providing unique information probing different aspects of the brain’s structural and functional organization. *Anatomical MRI* provides information about brain structure, most commonly the volumes and shapes of particular brain regions. *Diffusion Tensor Imaging (DTI)* provides information about tissue organization within the brain, especially white matter fibers that connect one brain region to another. At each voxel, Fractional Anisotropy (FA) measures the directional diffusion of water, Mean Diffusivity (MD) measures the overall diffusion of water, Axial Diffusivity (AD) measures diffusion along the long axis of diffusion, which is presumed to be along the long axis of the fiber bundle, and Radial Diffusivity (RD) measures diffusion perpendicular to the long axis of diffusion or fiber bundle. *Functional MRI (fMRI)* provides measures of time-dependent changes in the relative concentration of deoxyhemoglobin within each voxel, which itself is driven largely by changes in oxygen use and the underlying changes in neuronal activity within the voxel [87, 88]. Measures of *resting state connectivity (rsfMRI)* are in essence measures of cross-correlation for the fMRI series of two different voxels (i.e., two different points in the brain), which assesses how strongly those two regions communicate with one another over time.

##### MRI Scanning

All scans will be acquired on 3.0 T scanners: Siemens Prisma (Frankfurt, Lausanne), Philips Achieva (CHLA). Signal-to-noise and contrast-to-noise was determined to be comparable across all scanning platforms (Tables 4, 5, 6 and 7).

**Table 4:** Anatomical MRI

Scan Parameters for 3.0T MRI Scanners	Siemens	Philips
Coil	64	32
Sequence	MPRAGE	3D Gradient Echo Turbo Field Echo factor = 130
Scan plane	Sagittal	Sagittal
Direction of frequency coding	H-F	S/I
Echo time	2.75 ms	2.8 ms
Inversion Preparation Time	920 ms	920 ms
Flip Angle	9	9
Bandwidth	190 Hz/pixel	206 Hz/pixel
Repetition time (or MPRAGE TR)	2000 ms	3000 ms
Field of View	256 mm	256 mm
Matrix	256 × 256	256 × 256
Slice thickness	1.0 mm 176 slices	1.0 mm 176 slices
Phase Field of View	1.0	1.0
Number of excitations	2	2
Time	9 min, 20 secs	9 min, 3 secs

**Table 5:** Task-based fMRI: cyberball

Sequence	2D Gradient Echoplanar	2D Gradient Echoplanar
Scan plane	Axial Oblique, parallel to the AC-PC line	Axial Oblique, parallel to the AC-PC line
Effective Echo time	34 ms	34 ms
Flip Angle	90^o^	90^o^
Repetition time	4630 ms	4630 ms
Field of View	24 cm	24 cm
Matrix	112 × 112	112 × 112
Slice thickness	2.4 mm	2.4 mm
Number of Volumes/Run	215	137
In-Plane Resolution	24 cm/112 × 24 cm/112 = 2.14 × 2.14 mm2	24 cm/112 × 24 cm/112 = 2.14 × 2.14 mm2
Number of Slices	60	60
Number of Runs	1	1
Time	16 min 40 s (1000 s)	10 min 34 s (643 s)

**Table 6:** Resting-State fMRI

	**Siemens**	**Philips**
Sequence	2D Gradient Echoplanar	2D Gradient Echoplanar
Scan plane	Axial Oblique, parallel to the AC-PC line	Axial Oblique, parallel to the AC-PC line
Echo time	34 ms	34 ms
Flip Angle	90^o^	90^o^
Repetition time	4630 ms	4630 ms
Field of View	24 cm	24 cm
Matrix	112 × 112	112 × 112
Slice thickness	2.4 mm	2.4 mm
Number of Volumes/Run	90	90
In-Plane Resolution	24 cm/112 × 24 cm/112 = 2.14 × 2.14 mm2	24 cm/112 × 24 cm/112 = 2.14 × 2.14 mm2
Number of Slices	60	60
Number of Runs	1	1
Time	7 min 11 s (1000 s)	7 min 11 s (1000 s)

**Table 7:** Diffusion tensor imaging

	**Siemens**	**Philips**
Sequence	2D Gradient Echoplanar	2D, single-shot, echo-planar imaging
Scan plane	Axial Oblique, parallel to the AC-PC line	Axial Oblique, parallel to the AC-PC line
Echo time	66 ms	66 ms
Flip Angle	90^o^	90^o^
Repetition time	7300 ms	7700 ms
Field of View	24 cm	24 cm
Matrix	112 × 112	112 × 112
Slice thickness	2.0 mm	2.0 mm
Acceleration factor	2 (GRAPPA)	2 (ASSET)
Number of DWIs	64	64
In-Plane Resolution	24 cm/112 × 24 cm/112 = 2.14 × 2.14 mm^2^	24 cm/112 × 24 cm/112 = 2.14 × 2.14 mm^2^
Number of Slices	72	72
Number of Runs	2	2
b-values	0, 1000 s/mm^2^	0, 1000 s/mm^2^
Time	8 min 46 s	About 7 min

##### Theoretical basis for the fMRI task used in MODE

Chronically depressed patients need to experience an intensive, professional, reliable, holding, and containing therapeutic relationship in order to both regain confidence in a "helping object" in the form of the therapist and develop a basic feeling of self-agency. According to psychoanalytic theory, severe early life trauma produces a defensive withdrawal of the self from outside objects into a depressed inner world. Metaphorically speaking, the self is encapsulated in an inner crypt [89]. Therefore, as a first indispensable step in a therapeutic relationship, the withdrawn, depressed patient must regain confidence in an external object (i.e. the therapist) to dare to confront himself with the traumatic experiences in the transference relationship, in the hope of making alternative, "healing" experiences. The fMRI task we have selected and adapted for use in MODE is intended to index the development of this basic trust in others in response to the therapeutic interventions.

##### Task-based fMRI ‘Cyberball’ paradigm

MODE will study the neurobiological mechanisms that subserve this evolving sense of interpersonal trust within patients over the first year of psychoanalytic therapy. We have adapted a task that participants perform while in the MRI scanner so as to identify the brain systems that support the cognitive, emotional, and behavioral requirements of the task. This fMRI task, called “Cyberball” [90], is a computer game in which participants believe they are playing with real people in another room of the building. It is designed to manipulate the independent variable of social exclusion, via including and then suddenly excluding the participant in the ball-tossing. The game begins with the participant and two other “players” throwing a ball to one another, each of the three players receiving it an equal number of times. Without warning, the other two players then stop throwing the ball to the study participant, an experience that has been shown to produce feelings of betrayal and social exclusion. After some time, they once again throw the ball to the study participant, which produces feelings of relief upon re-inclusion. Brain activity during each of the three phases of this fMRI task will be measured at study baseline and after one year of psychoanalytic therapy [91–93]. Our aim is to identify the neural systems that support the expression of trust, mistrust, and social exclusion, and then reintegration of trust in interpersonal relationships, how activity in these systems change over the year in depressed patients compared with the change observed in healthy controls, and how high vs low frequency of PA therapy modifies activity in these systems over time.

#### Secondary outcome measures

MODE will investigate whether and how brain changes are associated with common measures of change for patients with chronic depression and early trauma after one year of treatment. To achieve this aim and as recommended in meta-analytic reviews, various broad areas of patient’s functioning are assessed including (1) psychopathology, (2) personality, social and work functioning, and (3) dynamic functioning which may underlie impaired functioning and contribute to vulnerability to psychiatric disorders [94]. Due to their depressive symptomatology, patients may face some difficulties with self-report questionnaires. Therefore, we have restricted the total number of items included in the assessment battery (Table 8).

**Table 8:** Secondary outcomes: clinical response measures

Clinical therapeutic outcomes	Clinical-psychoanalytical outcomes
• Beck Depression Inventory (BDI)	• Operationalized Psychodynamic Diagnostics self-report questionnaire (OPD-SQ)
• Depressive Experience Questionnaire (DEQ)	• The Social Cognition and Object Relations Scale-Global Rating Method (SCORS-G)
• Social Functioning Global Assessment of Functioning (GAF)	• Reports of trauma memories (Pennebaker)
• Work Ability Index (WAI)	
• Therapeutic alliance Working Alliance Inventory (WAI-SRp)	
• Inventory of Interpersonal Problems (IIP)	

##### Assessment of psychopathology

###### Diagnosis

The Structured Clinical Interview for DSM (SCID, [95]) is used to collect diagnostic information. This instrument enables the collection of extensive information on psychiatric conditions including mood and anxiety disorders.

###### Depression

Depressive symptoms will be assessed using three instruments: (1) The Inventory of Depressive Symptom (QIDS; [96]), a clinician rating measure (QIDS-C_16_); (2) the self-rated version of the QIDS (QIDS-SR_16_), 16-item measures of depressive symptoms; and (3) the Berg -Depression Inventory (BDI-II; [97]). The Depressive Experience Questionnaire (DEQ; [98]), which assesses a wide range of life experiences often reported by depressed individuals but not considered symptoms of depression.

###### Distress

The Symptom Check-List (SCL-90-R; [99]) is a widely used self-report measure of distress and psychiatric symptoms.

###### Early life trauma

Early childhood trauma is recognized as one of the best predictors of response to psychotherapy for severely depressed patients ( [100]). The Childhood Trauma Questionnaire Short-Form (CTQ-SF; [101]) provides a quick, multidimensional, retrospective measure of childhood trauma with good reliability and validity.

###### Patient trauma narratives

Patients will be asked to send a written trauma narrative to the study center with 5 keywords. After the year of study, the research team will send again the keywords to the participant and will ask them to write again their memories on trauma. The trauma narrative will be written freely, with the instruction to write about their deepest thoughts and feelings about the most disturbing aspects of their childhood. Participants will be asked to write during 15 to 20 min once started. The Linguistic Inquiry Word Count (LIWC; [102]) will be applied to patient trauma narratives to investigate patient narratives about their central early trauma as well as to their dream diaries [103, 104].

##### Dynamic personality functioning

###### Psychodynamic functioning and change

The Operationalized Psychodynamic Diagnosis (OPD) is a form of multiaxial diagnostic system based on five axes: I = experience of illness and prerequisites for treatment, II = interpersonal relations, III = conflict, IV = structure and V = mental and psychosomatic disorders ( [105]). To measure change in the domain of psychodynamic functioning, we will use the short-form of this questionnaire (OPD-SF, [106]).

###### Dream diary

Dreams will be collected in dream diaries ideally at each follow-up points and latest at study termination with the intent to examine dream characteristics in participants with chronic depression and early trauma. Participants can write freely about their dreams. To help participants document their dreams, an exemplary table will be provided where they can write a description of their dream(s) and report on the feelings they experienced in the dream(s) as well as elements of real life associated to the dream(s). Dream characteristics and changes of the manifest content of dreams as reported in the dream diaries will be assessed using the Social Cognition and Object Relations Scale-Global Rating Method (SCORS-G**;** [107]) as well as the LIWC (see above).

##### Psychosocial functioning

###### Interpersonal problems

The Inventory of Interpersonal Problems (IIP; [108]) is a self-report measure of problems in interpersonal relationships. It is widely used in psychotherapy research.

###### Interpersonal trust

Primal trust in a helping Other has been proposed as an important feature of therapeutic interaction [47]. We will use the Trust game to measure trust and reciprocity in participants [109]. This is a paradigm drawn from behavioral economics that tests how much money a person would give to a stranger in order to potentially receive a higher amount [110].

###### Therapeutic Alliance

Alliance plays an important role in effective psychotherapy [111]. Alliance with the individual therapist will be assessed using the patient self-rated Working Alliance Inventory Short-Form (WAI-SR; [112]).

###### The Global Assessment of Functioning (GAF)

Is a numeric scale (0 through 100) used by an independent coder to rate the social, occupational and psychological level of functioning [113]*.*

###### Work Ability Index (WAI-SR)

A previous study found that short- and long-term psychodynamic psychotherapy were differentially effective in improving work ability [114]. We will use the WAI to assess work ability, a questionnaire used in occupation health and research [115].

###### MRI Scanning MR spectroscopy (1H MRS)

Scans are acquired on 3.0 T scanners, Siemens Prisma in Frankfurt subgroup only. Spectroscopy was performed at the end of the MRI examination. An axial slice was recorded with 2D MRSI using an acquisition weighted circular phase encoding scheme on a 20 × 20 matrix, FOV of 240 × 240 mm^2^, 12 mm slice thickness, nominal voxel size of 12 × 12x12 mm^3^, TR 2000 ms, TE 40 ms, and 2 acquisitions. The volume of interest (VOI), which was selected by using the sLASER sequence from the vendor and outer volume suppression, was adjusted to contain GM and WM. Spectra were analyzed using LCModel [116]. The basis data set contained simulated spectra from Alanine, Creatine, GABA, Glucose, Glutamine, Glutamate, Glycine, Choline, Glutathion, Lactate, Mio-Inositol, N-acetyl-aspapartate-glutamate and N-Acetyl-Aspapartate.

## Procedures

### Data collection sites

Recruitment and treatment sites will be located in one center in the US (Los Angeles) and six centers in Europe (Germany: Frankfurt a.M., Cologne, Leipzig, Giessen, Mainz; and Switzerland: Lausanne). MRI scanning occurs in Los Angeles, Frankfurt a.M., and Lausanne. MRI data are processed in Los Angeles (CHLA). A clinical agreement has been signed and approved by each participating site.

### Patient recruitment

Patients will be recruited in private psychotherapeutic and psychiatric practices, in outpatient clinics for mood disorders in hospitals. Patient flyers describing study aims and procedures will be available to potential participants with a likely diagnosis of chronic depression. Healthy controls will be recruited through informal networks. Patients with a likely diagnosis of chronic depression (BDI-II > 17) and interested healthy volunteers will be referred to the respective study center in each country and thereafter interviewed for eligibility by a research assistant.

### Presentation of the study and informed consent procedures

The study will be presented to potential participants. Documents explaining the study, with its aims, procedures, benefits and risks for the participants will be provided to the participants. Interested subjects will be given a period to consider whether they would like to participate and the research team will be at their disposal to answer their eventual questions on their participation. Subjects confirming their interest in participating will then be asked to provide their informed consent to be randomly assigned to one of the treatment settings, to participate in the diagnostic, MRI, and psychometric procedures, to agree that some psychotherapy sessions are audio recorded, and agree to participate in the follow-up measurements). Participants with chronic depression and early life trauma will then be randomized in one of the two intervention groups.

### Randomization

The randomization procedure will consist of a computer-generated sequence of random numbers using SPSS (SPSS Statistics, version 28), which will then be used to allocate patients to either of the 2 treatment arms. The randomization sequence will be generated and maintained by an independent study administrator in Frankfurt. Once the patient is included the ID number is communicated to the study administration in Frankfurt. On the same day, the randomization arm will be communicated to the local team and applied to the participant included.

### Blinding

All the members of the investigating team (SCID /LIFE interviewers, MRI team etc.) will be blinded (they do neither know the treatment arm nor the names of the study therapists).

### Rater training

SCID interviewers will be masters-level psychologists, previously trained on administration of SCID and supervised be a senior study investigators.

### Manualized psychotherapeutic interventions

In the LAC study, therapists were trained in the specific treatment technique of psychoanalysis using the Tavistock Manual [117, 118], which we have modified and extended for the MODE study [119]. The MODE manual was developed by leading clinical experts of all participating sites of the study. It integrates various psychoanalytic traditions and cultures for treating chronically depressed, traumatized patients and describes high- and low-treatments. French and English translations are available.

### Therapist selection and training

Both interventions will be delivered by board certified psychoanalytic therapists which have trainings and clinical experiences in low frequent and high frequent psychoanalytic therapies. Trained therapists will deliver both treatments, depending on their availability and vacancies at the time of participant enrollment. All MODE therapists are trained in the Manual [119]. Most of the study therapists in Germany have already been trained and worked in the LAC Study. Additional training will be offered for them and the new MODE study therapists of all centers in several workshops. It will include theoretical presentations, group discussion of exemplary cases, and individual case presentations. Training will be conducted by expert trainers who are qualified psychoanalysts and authors or collaborators in developing the Manual.

### Clinical supervision

All study therapists will participate in monthly group supervisions or in one of the monthly “Clinical Conferences” (chairs in Germany: Marianne Leuzinger-Bohleber, Tamara Fischmann, in Lausanne: Gilles Ambresin, in SF/LA: Cheryl Goodrich), where therapists present their ongoing psychoanalyses with study patients.

### Recording and transcription

A random selection of therapy sessions will be audiotaped. Adherence to the treatment protocol will be assessed by independent members of the research teams using the Comparative Psychotherapy Process Scale (CPPS; [120], see also [29]).

### Power calculation

Power analyses using MRI measures from prior RCTs of medication therapy for the treatment of chronic depression [81, 82] indicated that a sample size of N = 30 participants for each of the 3 arms of the study would afford us a power of 80% to detect a moderate-to-large effect size (Cohen d = 0.5) with an α = 0.05 in each imaging modality (anatomical, task-based fMRI, resting state fMRI, and DTI).

### Data analysis

Analyses will follow an intent-to-treat strategy comparing patients in the groups to which they were originally randomized.

All data will be scored and rescored by separate staff members and checked for accuracy prior to double entry into a REDCap database. Before conducting inferential statistics, we will explore data using descriptive analyses, data visualization, and examining the distributions of all imaging and clinical data. When necessary for continuous variables used as dependent variables, appropriate transformations to obtain approximate normality will be performed.

We will test a priori Primary Hypotheses in the 60 depressed participants by assessing a treatment-by-time interaction in multiple general linear models with repeated measures analyses in an intent-to-treat analysis applied to our various brain measures as dependent variables, including cortical thickness, task-based fMRI activation, resting state connectivity, and FA & ADC measures. Treatment (high vs low frequency) will be the independent variable. When testing hypotheses, we will account appropriately for the correlation of repeated measures across the two time points of measurement (baseline and 1 year) and the spatial correlation within each of our imaging measures. Covariates will include age and sex of the participants. We will examine parameter estimates and 95% confidence intervals, approximate the p-values of component terms, and estimate effect sizes in our final models. Least squares estimates (or maximum likelihood estimates) and their standard errors will be calculated for all models and plotted to assist in interpretation of significant main effects and their interactions. We will ensure that overall, study-wide Type I error-rate is maintained, correcting for multiple comparisons using False Discovery Rate (FDR). FDR-corrected *p*-values < 0.05 will be color-coded and displayed on a template brain.

The 30 healthy controls will be used to estimate the mean and variance of brain measures in the healthy population quantify the effects of high and low frequency psychoanalysis on brain measures and to aid their interpretation as either normalizing a previously abnormal brain measure or introducing a new brain difference that attenuates symptoms.

#### Missing data

We will model missing responses/covariates in our longitudinal models. We will consider missing data mechanisms, including missing completely at random, missing at random, or missing not at random, to handle missing data due to a missed assessment time point, study dropout, or unusable MRIs. The full information maximum likelihood estimation (FIML) method will be used in fitting statistical models including LMM and LVM, which allow incomplete data (i.e., cases with missing data will contribute to the analysis).

## Discussion

The findings of MODE will be relevant for both clinical and basic research in identifying the brain-based mechanisms whereby psychotherapy exerts its therapeutic effects. MODE is, to our knowledge, the first study to investigate the neurobiological changes in response the effects of intensive psychoanalytic psychotherapy. MODE will thus contribute to the methodology of using MRI studies in translational psychotherapy. MODE may be the first in a series of studies combining neurobiological, psychological, and psychoanalytical approaches. It will allow us to establish outcome measures on a neural level that are perhaps more sensitive in distinguishing the differential effects of therapeutic dosing than established behavioral and clinical markers of symptom severity and change can provide.

MODE can also offer an answer to the question of why a relatively large group of patients with major depression respond poorly to treatment. Our initial studies suggest that these are patients who often suffered severe trauma before the age of 7 [51, 121]. MODE may help chronic depression patients with early trauma access a tailored treatment that addresses their specific needs. Because of the extremely high prevalence of depression in populations with early trauma, the development of effective and sustainable therapies for these difficult-to-treat patients is among the most urgent medical imperatives.

This research has a notable strength in its randomized controlled design. Yoking MRI to RCTs can identify neurobiological mechanisms predicting and causally mediating treatment response [80]. Most imaging study designs are associational and performed in samples of convenience. They therefore cannot support causal inferences for identifying disease etiology or treatment effects [80]. We hope to use the predictors that we discover to determine whether we can screen depressed patients for those biomarkers and thereby significantly improve treatment response rates in this highly treatment-resistant population. Identifying these markers will provide a framework for developing personalized care in depressed patients. Identifying the brain-based mediators of therapeutic response will support future efforts to enhance manipulation of those mediators to improve treatment response.

### Supplementary Information

**Additional file 1: Supplementary Table 1.** MODE study. Trial registration data.

## Data Availability

The datasets generated and/or analysed during the current study will be available on reasonable request to the PIs, via the corresponding author (gilles.ambresin@chuv.ch).

## Abbreviations

BDI-II: Beck Depression Inventory-II
CTQ: Childhood Trauma Questionnaire
HF: High Frequent treatment
IIP: Inventory of Interpersonal Problems
LAC: Langzeitbehandlungen chronisch Depressiver
LF: Low Frequent Treatment
MDE: Major Depressive Episode
MDD: Major Depressive Disorder
MODE: Multi-level Outcome study of psychoanalyses of therapy-refractory, chronically Depressed patients with Early trauma
MRI: Magnetic Resonance Imaging
NT: No Treatment
OPD: Operationalized Psychodynamic Diagnostics
PAT: Psycho-Analytic Treatments
QIDS: Quick Inventory of Depressive Symptoms
RCT: Randomized Controlled Trial
SCL: Symptom Checklist – 90
SCID: Structured Clinical Interview for DSM Disorders
WAI: Working Alliance Inventory
WAIx: Working Ability Index
