# Supplementary material for: The multi-level outcome study of psychoanalysis for chronically depressed patients with early trauma (MODE): rationale and design of an international multicenter randomized controlled trial
Source: BMC Psychiatry. 2023 Nov 16;23:844. doi: 10.1186/s12888-023-05287-6 (PMC10652457; doi:10.1186/s12888-023-05287-6)
Supplement: Supplementary file 1 — Additional file 1: Supplementary Table 1. MODE study. Trial registration data. [file 12888_2023_5287_MOESM1_ESM.docx]

**Supplementary Table 1 | MODE study. Trial registration data**

| **Data category** | **Information** |
| --- | --- |
| Primary registry and trial identifying number | DRKS00016872 |
| Date of registration in primary registry | 2019-03-12 Last Update 2023-01-30 |
| Secondary identifying numbers | U1111-1229-2321 |
| Source(s) of monetary or material support | International Psychoanalytic Association, London; American Psychoanalytic Association, New York, USA; Alfred Berman Foundation for Medical Research, USA; Robert S. Wallerstein Fellowship in Psychoanalytic Research, San Francisco Center for Psychoanalysis, USA; Deutsche Psychoanalytische Vereinigung, Berlin. |
| Primary sponsor | International Psychoanalytic Association, London |
| Secondary sponsor(s) | Prof. Marianne Leuzinger-Bohleber, Frankfurt, Germany; Prof Bradley Peterson, Los Angeles, California, USA |
| Contact for public queries | Prof. Dr. Tamara Fischmann [tamara.fischmann@ipu-berlin.de] |
| Contact for scientific queries | Prof. Marianne Leuzinger-Bohleber |
| Public title | Multimodal Outcome Study of Psychoanalyses of Chronically Depressed Patients with Early Trauma |
| Scientific title | *Multimodal Outcome Study of Psychoanalyses of Chronically Depressed Patients with Early Trauma* |
| Countries of recruitment | Germany, USA, Switzerland |
| Health condition(s) or problem(s) studied | Chronic depression with early trauma |
| Intervention(s) | *Active comparator I:* Psychoanalytical longterm treatment with 1 weekly session |
|  | *Active comparator II:* Psychoanalytical longterm treatment with 3-4 weekly session |
| Observational group | Healthy control group |
| Key inclusion and exclusion criteria | Ages eligible for study: 21-60; Sexes eligible for study: both; Accepts healthy volunteers: yes |
|  | *Inclusion criteria:* Diagnosis of major depression and/or dysthymia (based on Structural Clinical Interview-SCID) for at least 12 months, BDI-2 > 17, QIDS > 9, CTQ: at least trauma at one of the subscales, Age: 21-60 years, Sufficient knowledge of local language, Informed consent to study protocol, Need to be free of anti-depressant, anti-anxiety, and antipsychotic medication for at least 3 weeks before baseline assessment and be deemed stable by their treating clinician at the study onset. |
|  | *Exclusion criteria:* Current or past psychotic symptoms in the last 3 years, schizoaffective, schizophrenic, or bipolar affective disorder, Substance dependence current or during the last three years, Dementia, Borderline, schizotypal, or antisocial personality disorder, Acute suicidality, Reduced intellectual capacity, Serious physical illness that strongly affects the depression or is causal for the depression, Concurrent psychotherapy, Technical exclusion criteria for MRI (metallic tattoos, pacemakers, or other metal parts in the body etc., according to special guidelines of the local MRI team) |
| Study type | Interventional, Prospective |
|  | Allocation: randomized; Intervention model: parallel assignment; Masking: single blind (assessor, data analyst). |
|  | Primary purpose: Treatment |
|  | Phase N/A |
| Date of first enrolment | 2019-10-01 |
| Target sample size | N=90 |
| Recruitment status | Recruiting ongoing |
| Primary outcome(s) | Neurobiological Outcome measure (MRI) |
| Key secondary outcomes | Psychoanalytical Outcome measures: structural changes (as measured by the OPD SF (SR) and the Self Reflecting Functioning Scales)  Psychological Outcome measures: symptomatic and psychological changes (as measured by BDI-II (SR), QIDS (Cl., SR) and GAF (Cl), DEQ(SR), SCL-90-R (SR, II-P (SR)) |
